# Non-pharmacologic Treatment of Heavy Menstrual Bleeding in Persian Medicine

**DOI:** 10.31661/gmj.v9i0.1725

**Published:** 2020-06-24

**Authors:** Fatemeh Yousefi, Jale Aliasl, Fataneh Hashem-Dabaghian

**Affiliations:** ^1^Research Institute for Islamic and Complementary Medicine, School of Persian Medicine, Iran University of Medical Sciences, Tehran, Iran; ^2^Traditional Medicine Clinical Trial Research Center, Shahed University, Tehran, Iran

**Keywords:** Herbal Therapy, Heavy Menstrual Bleeding, Hypermenorrhea, Complementary Therapies, Traditional Medicine, Persian Medicine

## Dear Editor,

Menorrhagia is abnormal heavy or prolonged vaginal bleeding with 19.24% prevalence in Iranian women [1]. HMB can be diagnosed based on excessive (more than 80 ml) or prolonged (more than 7 days) uterine bleeding per menstrual cycle [2]. The cause of HMB may be structural and nonstructural problems [2]. There are nine main categories, arranged according to the acronym PALM -COEIN (polyp, adenomyosis, leiomyomas, malignancy, hyperplasia, coagulopathy, ovulatory dysfunction, endometrial disorders, iatrogenic causes and not-yet-classified entities) [2]. Severe anemia is the most important problem of HMB that induced signs and symptoms such as fatigue, headache, palpitation, dizziness, pallor and pica [3] and decreases the quality of life in women [4]. There are medical treatments to decrease bleeding including antifbrinolytics, nonsteroidal anti-inﬂammatory drugs, hormone therapy and tranexamic acid [5]. Although these drugs have effect on bleeding, but their several adverse reactions such as cerebrovascular infarction, myocardial infarction and pulmonary embolism are also significant [5]. Due to the side effects of medications, finding non-pharmacological and effective treatments such as complementary and traditional medicine is essential. In Persian medicine, Non-pharmacological treatments including nutrition modification and dry cupping recommended for management of HMB [6-9]. In fact, nutrition plays a very important role in the menstruation management and anemia caused by it [10]. In PM view point astringent food, such as lentil *( Lens culinaris)*, quince ( *Cydonia oblonga)* fruit paste, apple ( *Malus domestica )* paste, rhubarb ( *Rheum rhabarbarum )* paste, barberry ( *Berberis vulgaris)* paste, pomegranate ( *Punica granatum)* paste, bird’s meat with sumac * (Rhus* coriaria), lentil, barberry and almond ( *Prunus dulcis)*, *Purslane (Portulaca oleracea)* and coriander ( *Coriandrum sativum)* can decrease bleeding [6-9]. Eating lentil, especially with vinegar, is effective in chronic uterine bleeding [6,7]. In PM, some nutrients can increase blood production in the body. Therefore, their consumption are useful for improvement anemia. These foods include: chicken, lamb, kebab, broth, half boiled egg, milk, grape ( *Vitis vinifera)* syrup, white mulberry ( *Morus alba)* and fig ( *Ficus carica )* [6-9]. Nutrition can have negative effects on diseases. On the other hand, consumption of some food can increase vaginal bleeding that should be avoided [6-9]. Therefore, patients with HMB should be avoided these foods including: cabbage ( *Brassica oleracea var. capitate)*, red beans ( *Phaseolus vulgaris)*, sesame ( *Sesamum indicum)*, onion ( *Allium cepa)*, celery ( *Apium graveolens)*, turnip ( *Brassica rapa subsp. Rapa)*, carrot ( *Daucus carota subsp. Sativus)*, *garden cress (Lepidium sativum), *radish ( *Raphanus raphanistrum subsp. Sativus)*, ginger ( *Zingiber officinale)*, fenugreek ( *Trigonella foenum -graecum)*, cinnamon ( *Cinnamomum verum)*, black seeds ( *Nigella sativa)*, date ( *Phoenix dactylifera),* pennyroyal * (Mentha pulegium )* and bitter and spicy foods [6-9]. In addition to nutrition, one of the non-pharmacological methods for treatment of HMB is dry cupping [6-9]. In this way, the glasses are placed just below each breast and the vacuum was created with a mechanical suction pump for reducing the menstrual bleeding [6,7]. Ibn Sina believes that the dry cupping below the breast can moves substances including blood, from the uterus and can lead to reduced menstrual bleeding [7]. On the other hand Razi believed the uterus vessels is associated to the breast vessels therefore for stopping excessive menstrual bleeding, you should put cups below the breasts [6]. Various mechanisms have been proposed to reduce the severity of menstrual bleeding with cupping. One of the mechanisms is the change in endometrial vascular homeostasis [11,12]. Cupping affects the endometrial vascular homeostasis with various mechanisms such as suppressing vasodilator (prostaglandins), increasing platelet aggregation, thromboxane, and PGF2a (vasoconstrictors) [13,14]. Clinical studies confirm the efficacy of the cupping to reduce menstrual bleeding [13,15]. Recommended dietary plan and dry cupping based on Persian Medicine can be effective in women with HMB. Clinical studies was suggested for assessment of the above dietary plan and dry cupping on women with HMB.

## Acknowledgement

 This study was a part of the Ph.D. thesis of Fatameh Yusefi. Fataneh Hashem-Dabaghian was the supervisor and Jaleh Aliasl contributed to the writing the manuscript.

## Conflict of Interest

 The authors declare that there is no conflict of interests.
